# Complete genome sequence of a *Pseudomonas fluorescens* bacteriophage UNO-G1W1 isolated from freshwater ice in Nebraska

**DOI:** 10.1128/mra.00384-24

**Published:** 2024-06-07

**Authors:** Thomas T. Schulze, Andrew J. Neville, Gabrielle F. Watson, Austin G. Sanford, Harim I. Won, Mackenzie E. Conrin, Connor G. Eastman, LeeAnna M. Lui, M. Yunos Alizai, Matthias J. Walters, Paul H. Davis, William E. Tapprich

**Affiliations:** 1 Department of Biology, University of Nebraska at Omaha, Omaha, Nebraska, USA; 2 Department of Pathology, Microbiology, and Immunology, University of Nebraska Medical Center, Omaha, Nebraska, USA; Department of Biology, Queens College, Queens, New York, USA

**Keywords:** bacteriophages, *Pseudomonas fluorescens*, *Pseudomonas*, genomics, genomes, long-read seqeuncing, Oxford Nanopore, DTR, hybrid assembly

## Abstract

We provide the complete genome sequence for a novel *Pseudomonas fluorescens* bacteriophage named UNO-G1W1. This phage was isolated from a single ice cover sampling. The genome was sequenced on the Nanopore MinION, generated with the direct terminal repeat-phage-pipeline and polished with Illumina short reads. Sequence identity classifies the phage as an *otagovirus*.

## ANNOUNCEMENT

*Pseudomonas fluorescens* is a gram-negative bacterium commonly found in water and soil that is known for its versatile metabolome. While primarily described as a plant-promotive, nonpathogenic microbe, it has been associated with rare cases of bacteremia in humans (1). This effort sought to examine the feasibility of recovering viable bacteriophages in local freshwater resources with ice cover sampling, using *P. fluorescens* as the target host organism. The bacteriophage UNO-G1W1 was extracted by auger drill ice sampling from Lake Wanahoo at global positioning system (GPS) coordinates 41.251597 N, −96.612549 W (Wahoo, Nebraska, USA) on 20 December 2016. The thawed sample was filtered (0.22 µm), diluted, and added to log phase host bacteria (*P. fluorescens* Migula strain; ATCC 27663) at room temperature for 20 minutes for viral invasion. These bacteria were subsequently added to 0.7% agarose (45°C), plated and incubated overnight at 26°C. A single plaque was isolated and purified twice, and morphology was stable. A high titer stock was used to check purity via transmission electron microscopy (TEM) and to isolate genomic DNA by phenol-chloroform extraction as described previously (2).

A short-read library was prepared with the Illumina Nextera XT kit and sequenced on a Hi-Seq 2500 with 151 bp paired-end reads. FastQC 0.11.9 was used to verify read quality (http://www.bioinformatics.bbsrc.ac.uk/projects/fastqc/). A long-read library was prepared with the Oxford Nanopore Technologies ligation kit (SQK-LSK109). Briefly, to enrich for and improve recovery of long fragments following adapter ligation, a Long Fragment Buffer was used for washes, and incubation with Elution Buffer was carried out at 37°C. Sequencing was performed with a MinION Mk1B device using a FLO-MIN106D (R9.4.1) flow cell and MinKNOW software (v23.11.7). Live super-accurate basecalling was performed by Dorado (v7.2.13). A sequencing summary is provided in Table 1.

**TABLE 1:** Sequencing summary

Read library	Raw/total reads	Mean Q-score^*a*^	Total bases	*N*_50_ (bases)^*d*^	Mean/median read length (bases)^*d*^	Estimated seq. depth^*b*^	Mean depth of coverage^*c*^
Illumina R1	543,186	37.4	70.9 Mb	--	--	719×	1,349×
Illumina R2	543,186	36.7	70.9 Mb	--	--	719×	
Nanopore trimmed All reads	451,635	Mean: 7.9 Median: 10.0	7.44 Gb	31,888	Mean: 16,467 Median: 9,726	75,448×	64,869×
Nanopore assembly Reads (trimmed Q ≥ 10)	230,572	Mean: 12.3 Median: 13.2	4.08 Gb	33,491	Mean: 17,714 Median: 10,888	41,435×	40,604×

^*a*^ Illumina Q-scores calculated with BioPython and NumPy, Nanopore Q-score by NanoPlot v1.42.0 (3).

^*b*^ Total bases divided by complete genome size (98,572 bp).

^*c*^ Average depth by position calculated using SAMtools (v1.19.2, 4) Burrow-Wheeler Aligner (BWA, v0.7.17-r1188, 5) used to map Illumina reads. Minimap2 (v2.28-r1209, 6) used to map Nanopore reads.

^*d*^ Fields with “--” indicate a value that was not applicable or calculated.

To construct a complete genome, long reads were first filtered with a Q-score threshold of 10, and adapters were trimmed using PoreChop_ABI v0.5.0 (7). The direct terminal repeat (DTR)-phage-pipeline (https://github.com/nanoporetech/DTR-phage-pipeline, original release acc. 15 March 2024; default parameters, except “MEDAKA:model” as “r941_min_sup_g507”) was used to generate a polished long-read consensus genome (8). This sequence was further polished with Polypolish [v0.6.0 (9)], using Illumina short reads which revised a single single nucleotide polymorphism (SNP) in a low complexity region. The provided genome has a total length of 98,572 bp and guanine-cytosine (GC) content of 48.3%. Annotation was performed using Pharokka v1.7.1 and corresponding v1.4.0 database (10), which predicted 209 genomic features. These included 18 tRNAs and 132 predicted as hypothetical or proteins of unknown function (11, 12).

The DTR-phage-pipeline predicted the presence of a 544 bp DTR, but no clear evidence of circular permutation was detected. Whole genome BLASTn (22 March 2024) identified *P. fluorescens* bacteriophage phiPsa374 (accession: NC_023601.2) as the closest relative with 84.62% sequence identity and 63% coverage. Based on identity and recent taxonomic revisions (13), this novel bacteriophage belongs to the *Caudoviricetes* class (NCBI: txid2731619) and *Otagovirus* genus (NCBI: txid2560197).

## Data Availability

The complete genome sequence is available on NCBI GenBank as accession PP551948. The version described in this paper is the 2nd version, PP551948.2. Illumina and Nanopore raw reads are available from SRA accessions SRR28523409 and SRR28523408, respectively.
